# Boron Oxide Nanoparticles Exhibit Minor, Species-Specific Acute Toxicity to North-Temperate and Amazonian Freshwater Fishes

**DOI:** 10.3389/fbioe.2021.689933

**Published:** 2021-05-28

**Authors:** Tyson J. MacCormack, Patrick T. Gormley, B. Ninh Khuong, Olivia A. Adams, Susana Braz-Mota, Rafael M. Duarte, Christopher M. Vogels, Luc Tremblay, Adalberto L. Val, Vera M. F. Almeida-Val, Stephen A. Westcott

**Affiliations:** ^1^Department of Chemistry and Biochemistry, Mount Allison University, Sackville, NB, Canada; ^2^Laboratory of Ecophysiology and Molecular Evolution, Brazilian National Institute for Research of the Amazon, Manaus, Brazil; ^3^Institute of Biosciences, São Paulo State University (UNESP), São Vicente, Brazil; ^4^Department of Chemistry and Biochemistry, Université de Moncton, Moncton, NB, Canada

**Keywords:** nanotoxicology, engineered nanomaterials, acetylcholinesterase, ionoregulation, energy metabolism, oxidative stress, aquatic toxicology

## Abstract

Boron oxide nanoparticles (nB_2_O_3_) are manufactured for structural, propellant, and clinical applications and also form spontaneously through the degradation of bulk boron compounds. Bulk boron is not toxic to vertebrates but the distinctive properties of its nanostructured equivalent may alter its biocompatibility. Few studies have addressed this possibility, thus our goal was to gain an initial understanding of the potential acute toxicity of nB_2_O_3_ to freshwater fish and we used a variety of model systems to achieve this. Bioactivity was investigated in rainbow trout (*Oncorhynchus mykiss*) hepatocytes and at the whole animal level in three other North and South American fish species using indicators of aerobic metabolism, behavior, oxidative stress, neurotoxicity, and ionoregulation. nB_2_O_3_ reduced *O. mykiss* hepatocyte oxygen consumption (*Ṁ*O_2_) by 35% at high doses but whole animal *Ṁ*O_2_ was not affected in any species. Spontaneous activity was assessed using *Ṁ*O_2_ frequency distribution plots from live fish. nB_2_O_3_ increased the frequency of high *Ṁ*O_2_ events in the Amazonian fish *Paracheirodon axelrodi*, suggesting exposure enhanced spontaneous aerobic activity. *Ṁ*O_2_ frequency distributions were not affected in the other species examined. Liver lactate accumulation and significant changes in cardiac acetylcholinesterase and gill Na^+^/K^+^-ATPase activity were noted in the north-temperate *Fundulus diaphanus* exposed to nB_2_O_3_, but not in the Amazonian *Apistogramma agassizii* or *P. axelrodi*. nB_2_O_3_ did not induce oxidative stress in any of the species studied. Overall, nB_2_O_3_ exhibited modest, species-specific bioactivity but only at doses exceeding predicted environmental relevance. Chronic, low dose exposure studies are required for confirmation, but our data suggest that, like bulk boron, nB_2_O_3_ is relatively non-toxic to aquatic vertebrates and thus represents a promising formulation for further development.

## Introduction

When released into the environment, bulk boron compounds and boric acid (BA) can decompose into nanoparticles (NPs) (Zhou and Bai, 2015). Boron NPs are also manufactured as engineered nanomaterials (ENMs) and boron ENMs in the form of boron oxide (nB_2_O_3_) are used in fuel and propellant applications for their high heats of combustion and rapid energy release rates (Liu et al., 2010). As nB_2_O_3_ use increases, more will inevitably end up in the environment and could pose risks to human and ecosystem health. The toxicity of bulk boron in the form of BA has been well established in a number of terrestrial and aquatic organisms (Weir and Fisher, 1972; Butterwick et al., 1989; Cochran, 1995), however little is known about the toxicity of nB_2_O_3_. The only available study suggested that nB_2_O_3_ was more toxic to a model invertebrate than titanium dioxide and aluminum oxide ENMs (Strigul et al., 2009). nB_2_O_3_ is unlikely to find its way into the environment in the same quantities as more common ENM formulations (e.g., silver ENMs), but understanding its potential ecotoxicity remains important, especially if its use increases.

Boric acid is a natural form of boron found in minerals such as boracite, ulexite, and colemanite, as well as in seawater and plants (Lodge, 1994; Wolska and Bryjak, 2013), and is used in a variety of applications including clinical products, antibacterial and antifungal agents, and insecticides (Siegel and Wason, 1986; Sander et al., 1991; Xue and Barnard, 2003). Boron deficiency limits growth in many plants and the agricultural application of boron is common, particularly in some areas of Brazil where soil boron levels are sub-optimal (Fageria and Nascente, 2014). Boron is also an effective pesticide since it is essentially inert to mammals (Weir and Fisher, 1972) but is highly toxic to invertebrates (Cochran, 1995). For example, in German cockroaches, BA exhibits neurotoxicity by inhibiting acetylcholinesterase (AChE) activity and disrupting reactive oxygen species metabolism, as evidenced by increased glutathione S-transferase activity (Habes et al., 2006). In fish, boron does not bioaccumulate and toxicity depends on differences in osmoregulatory function, water uptake rates, membrane permeability, and environmental salinity (Thompson and Davis, 1976). Boric acid is relatively non-toxic to chinook and coho salmon, with 96 h LC50s > 100 mg L^–1^ (Hamilton and Buhl, 1990). At low concentrations, boron can actually be beneficial to fish; at <925 μmol L^–1^, BA stimulated embryonic growth in rainbow trout (Eckhert, 1998) and similar effects have been observed in zebrafish (Rowe and Eckhert, 1999).

Numerous ENM formulations exhibit some degree of acute toxicity to freshwater fish, although usually at exposure concentrations far in excess of those expected in the environment (Callaghan and MacCormack, 2017). Some commonly observed effects of acute ENM exposure are disruptions to ionoregulatory homeostasis (Schultz et al., 2012), oxidative stress (Federici et al., 2007), inhibition of AChE (Callaghan et al., 2016), and damage to the gill epithelium (Griffitt et al., 2009; Bessemer et al., 2015). Direct and/or indirect effects on energy metabolism at the tissue and whole animal level have also been observed (Dieni et al., 2014; Callaghan et al., 2016), in some cases at environmentally relevant ENM concentrations (Black et al., 2017). Under aerobic conditions, these effects can manifest as changes in rates of oxygen consumption (*Ṁ*O_2_) at either the cell or whole animal levels. At the whole animal level, routine and maximum *Ṁ*O_2_ (*Ṁ*O_2m__*in*_ and *Ṁ*O_2m__*ax*_, respectively) and aerobic scope, the difference between them, are ecologically relevant indicators of health and fitness (Brown et al., 2004). Contaminant-induced reductions in aerobic scope can have negative consequences on fish populations (Johansen and Esbaugh, 2017). ENM exposure influences whole animal *Ṁ*O_2_ in fish under certain conditions (Callaghan et al., 2016), sometimes in the absence of clear biochemical indicators of toxicity (Black et al., 2017; Campbell et al., 2019). Understanding how nB_2_O_3_ exposure affects aerobic metabolism in a variety of fish species will contribute valuable information on its overall environmental safety.

Thus, the present study had two objectives: the first was to characterize the impact of BA and nB_2_O_3_ on aerobic metabolism in isolated rainbow trout (*Oncorhynchus mykiss*) hepatocytes as a proxy for cellular heath. Lowest observable effects concentrations (LOECs) from these experiments, or 48 h LC_50_ trials, were then used to characterize the *in vivo* impacts of acute nB_2_O_3_ exposures on a common north-temperate fish, *Fundulus diaphanus*, and two Amazonian species, *Apistogramma agassizii* and *Paracheirodon axelrodi*. We have previously illustrated that these Amazonian species exhibit different sensitivities to ENM exposure as a result of their differing ionoregulatory and metabolic strategies (Braz-Mota et al., 2018), so they represent a powerful model system for studying the potential toxicity of nB_2_O_3_. Whole animal aerobic metabolic rates were determined, along with various biochemical indicators of toxicity to gain insight into potential mechanisms of nB_2_O_3_ bioactivity.

## Materials and Methods

### Nanoparticles and Characterization

Spherical B_2_O_3_ nanopowder (#1303862) was purchased from American Elements (Los Angeles, CA, United States) with advertised diameters of 20–80 nm and a specific surface area of 10–50 m^2^ g^–1^. Supplemental scanning electron microscopy (SEM) analysis was carried out to confirm primary particle size and shape. Dry nB_2_O_3_ was dusted onto carbon adhesive tabs (Cat. #77825-09, Electron Microscopy Sciences, Hatfield, PA, United States) affixed to aluminum stubs and sputter coated with ca. 5 nm gold using a Hummer 6.2 sputtering system (Anatech United States, Union City, CA, United States). Preparations were examined using a Hitachi SU3500 scanning electron microscope (Hitachi High Technologies, Toronto, Canada) operating at 5 kV and 5 mm working distance. The non-conductive and magnetic nature of the material prevented the use of lower energy beams for improved surface resolution. Morphological measurements of particles were manually acquired from SEM images with dmfMeasure, an image analysis program developed at Mount Allison University’s Digital Microscopy Facility.

Hydrodynamic diameter and zeta (ζ) potential were assessed via dynamic light scattering analysis using a Zetasizer Nano ZS (Malvern Panalytical, Malvern, United Kingdom) according to the recommendations of the instrument manufacturer. Fresh nB_2_O_3_ stock suspensions (100 mg L^–1^) were prepared in 0.2 μm-filtered well water from Mount Allison University’s Harold Crabtree Aqualab (Sackville, New Brunswick, Canada) or the Instituto Nacional Pesquisas de Amazônia (INPA; Manaus, Amazonas, Brazil). The composition of individual well waters has been described elsewhere (Bessemer et al., 2015; Braz-Mota et al., 2018) but additional characterization of the dissolved organic carbon (DOC) concentration in Mount Allison University’s well water was obtained. Measurements were carried out on filtered and acidified (pH 2 with HCl) samples with an Elementar isoTOC high-temperature catalytic oxidation analyzer (Elementar Americas Inc., NY, United States). Stock nB_2_O_3_ suspensions were sonicated for 30 s using a wand-type sonicator (F60 Sonic Dismembrator, Fisher Scientific, Ottawa, Canada) immediately prior to analysis.

### Animal Collection and Housing

Rainbow trout (*Oncorhynchus mykiss*; body mass 600 ± 25 g) were obtained from Fraser’s Mills Fish Hatchery (Nova Scotia, Canada) and banded killifish (*Fundulus diaphanus*; body mass 8.29 ± 0.28 g) were captured by hand netting from a local pond in Sackville, New Brunswick, Canada. Both species were housed in Mount Allison University’s Harold Crabtree Aqualab at 16 ± 1°C in separate 750 L tanks supplied with aerated, flow-through well water. The dwarf cichlid (*Apistogramma agassizii*; body mass 0.320 ± 0.018 g) and cardinal tetra (*Paracheirodon axelrodi*; body mass 0.161 ± 0.010 g) were purchased from an ornamental fish supplier in Manaus and housed at INPA, where they were maintained at 28 ± 1°C in 450 L tanks of aerated, flow-through well water. All animals were adults. Throughout the acclimation period, all fish were fed daily with dry commercial trout pellets and were kept on a 12 h light, 12 h dark photoperiod. All animals were allowed to acclimate for at least 2 weeks before the start of experiments and food was withheld 24 h before and during experiments. Procedures were approved by the Mount Allison University Animal Care Committee (protocols 15–15 and 101877). For Amazonian fish, procedures followed the CONCEA (National Council of Animal use in Research and Education) animal care guidelines and were approved by INPA’s animal care committee (protocol number: 026/2015).

### Hepatocyte Isolation and Cellular *Ṁ*O_2_ Measurements

Initial bioactivity testing was carried out on *O. mykiss* hepatocytes extracted following established protocols (Moon et al., 1985) with minor modifications. Fish were anesthetized with tricaine methanesulfonate (MS222; 300 mg L^–1^) buffered with NaHCO_3_ (600 mg L^–1^) and euthanized by severing the spinal cord. The hepatic vein was perfused with medium containing (in mmol L^–1^) NaCl (176), KCl (54), MgSO_4_ (0.81), KH_2_PO_4_ (0.44), Na_2_HPO_4_ (0.35), NaHCO_3_ (5.0), HEPES (10.0) and EGTA (1.0), pH 7.63. After 10 min, the liver was excised and transferred onto a watch glass containing the above medium but with added 0.3 mg mL^–1^ type II collagenase (#9001121, Sigma-Aldrich, United States) and no EGTA. After 30 min, the liver was gently teased apart and the suspension was repeatedly pipetted to break up cell aggregates. Cells were then filtered through a 100 μm nylon mesh, washed three times with perfusion medium containing 2% BSA and 1.5 mmol L^–1^ CaCl_2_, and centrifuged at 1,000 × *g* for 10 min. Isolated cells were resuspended in Ca^2+^-free perfusion medium and counted with a Neubauer hemocytometer. Cell viability was assessed by Trypan blue exclusion and preparations with viabilities <85% were rejected. Cell suspensions were supplied with 99.5% O_2_ and 0.5% CO_2_ and kept on ice prior to measuring *Ṁ*O_2_.

Hepatocyte *Ṁ*O_2_ was measured using OX1LP Dissolved O_2_ Cuvette Electrodes (Qubit Systems Inc., Kingston, ON) interfaced to Logger Pro Software via a LabQuest Mini data acquisition system (Vernier Software and Technology, Beaverton, OR, United States). Stock solutions of BA and nB_2_O_3_ were prepared in isolation medium and the nB_2_O_3_ stock solution was sonicated using a wand-type F60 sonic dismembrator (Fisher scientific, Waltham, MA, United States) for 30 s prior to use. Temperature was maintained at 16 ± 0.1°C using a recirculating water bath and cell suspensions were stirred throughout the experiment. 0.5 mL of cell suspension was transferred into each of the two cuvettes, one with additional isolation medium (control) and one with nB_2_O_3_ to final concentrations 0.1, 1.0, and 10.0 mg L^–1^. A triplicate *Ṁ*O_2_ measurement using aliquots of cell suspension from multiple independent cell isolations was done for each concentration. The average number of cells (16.13 × 10^4^ cells) in the cuvette (0.6 mL) was used to normalize *Ṁ*O_2_ to nmol O_2_ min^–1^ 10^4^ cells^–1^.

### *In vivo* Exposures

For *F. diaphanus*, nB_2_O_3_ exposures were carried out in a 30 L static reservoir of continuously aerated well water maintained at 16 ± 1°C. This species was exposed to a final concentration of 1.0 mg L^–1^ nB_2_O_3_ based on the results of the hepatocyte studies described above. Seven fish were placed in the tank and allowed to acclimate overnight prior to the addition of nB_2_O_3_ in the form of a freshly prepared and sonicated stock suspension. For control fish, an equivalent volume of ddH_2_O was added to the tank as a sham treatment. Animals were held for 48 h following nB_2_O_3_ or sham treatment before tissue sampling (see below). The relatively large volume of the experimental tank relative to the biomass of fish made a water change unnecessary, as ammonia did not increase above background levels during the exposure (data not shown).

Initial studies on *A. agassizii* and *P. axelrodi* focused on establishing a 48 h LC_50_ concentration for nB_2_O_3_. Static renewal exposures were carried out in glass aquaria containing 2 L of continuously aerated INPA well water at 28 ± 1°C, with 10 fish per aquarium. Fish were allowed to acclimate overnight, after which 50% of the water was changed and nB_2_O_3_ was added to final concentrations of 0, 0.1, 1.0, 10, and 100 mg L^–1^. A second 50% water change including the appropriate concentration of nB_2_O_3_ was carried out 24 h later and animals were monitored up to 48 h exposure.

At the end of the exposure period, the fish were anesthetized with MS222 buffered with Na_2_CO_3_. The fish were euthanized by severing the spinal cord and tissue samples were collected, flash frozen in liquid nitrogen, and stored at -80°C until use. For *F. diaphanus*, brain, heart, liver, and gill samples were collected, and for *A. agassizii and P. axelrodi*, gill and whole body samples were collected. For *A. agassizii* and *P. axelrodi*, only fish from the control and 10 mg L^–1^ nB_2_O_3_ exposure groups were used for biochemical analyses.

### Whole Animal Oxygen Consumption Measurements

The effect of nB_2_O_3_ exposure on aerobic metabolism *in vivo* was assessed by measuring *Ṁ*O_2_ in whole animals. For *F. diaphanus*, exposures were carried out on individual fish housed in a 140 mL intermittent flow respirometry chamber (Q-box Aqua, Qubit Systems, Kingston, Ontario, Canada) within the same 30 L static reservoir of continuously aerated well water maintained at 16 ± 1°C described above. Animals were placed in the respirometer in the afternoon and allowed to recover overnight before being exposed to 1.0 mg L^–1^ nB_2_O_3_ or a sham control. Routine *Ṁ*O_2_ (*Ṁ*O_2m__*in*_) was then determined in undisturbed fish up to 46 h post-exposure, after which fish were moved to a 1 L beaker of exposure water and manually chased to exhaustion (Healy et al., 2017). When *F. diaphanus* no longer responded to stimulation, they were quickly returned to the respirometer to measure maximum *Ṁ*O_2_ (*Ṁ*O_2m__*ax*_) as described elsewhere (Black et al., 2017).

Based on the results of the LC_50_ testing, *A. agassizii* and *P. axelrodi* were exposed to 10 mg L^–1^ nB_2_O_3_ or a sham control. Exposures were carried out on 6 fish simultaneously using the same static renewal protocol described above for LC_50_ testing. Immediately following the 48 h exposure, animals were transferred to a multi-channel intermittent flow respirometry system (Oxy-4; Loligo Systems, Vidborg, Denmark) and left undisturbed for a minimum of 12 h in clean well water. *Ṁ*O_2m__*in*_ was then quantified as previously described for these species (Braz-Mota et al., 2018). The layout of the multi-channel respirometry system precluded accurate measurements of *Ṁ*O_2m__*ax*_. The individual *A. agassizii* and *P. axelrodi* used for *Ṁ*O_2m__*in*_ measurements were not sampled for biochemical analysis.

### Biochemical Procedures

Lactate levels were measured in all species as an indicator of anaerobic stress. For *F. diaphanus*, lactate was measured in liver, while in *A. agassizii* and *P. axelrodi*, it was assessed in whole body homogenates prepared as previously described (Braz-Mota et al., 2018). *Fundulus diaphanus* liver samples were homogenized in 2% perchloric acid, centrifuged at 10,000 × *g* for 10 min, and the supernatant collected for analysis. In all instances, lactate was quantified by following the reduction of NAD^+^ to NADH by lactate dehydrogenase (LDH) at 340 nm in a glycine-hydrazine buffer and referenced to an L-lactic acid standard curve.

For *F. diaphanus*, lipid peroxidation and glucose-6-phosphate dehydrogenase (G6PDH) and glutathione reductase (GR) activities were used as indicators of oxidative stress in liver. Hepatic lipid peroxidation in *F. diaphanus* was quantified as malondialdehyde (MDA) using a commercially available kit (Bioxytech^®^ MDA-586, Oxisresearch, Berlingame, CA, United States) according to the manufacturer’s instructions. The activities of liver G6PDH and GR were measured using colorimetric assays that followed changes in NADPH absorbance at 340 nm (Dieni et al., 2014) and an extinction coefficient of 6.22 mM^–1^ cm^–1^. Oxidative stress assessments in *A. agassizii* and *P. axelrodi*, were carried out on whole body homogenates as previously described (Braz-Mota et al., 2018). In these species, lipid peroxidation was quantified using the colorimetric assay described by Jiang et al. (1991) and glutathione S-transferase (GST) activity was measured according to Keen et al. (1976). For GST assays, changes in 1-chloro-2,4-dinitrobenzene absorbance at 340 nm were converted to activities using an extinction coefficient of 9.6 mM^–1^ cm^–1^ and normalized to protein content using a Bradford assay (Bio-Rad Laboratories, Hercules, CA, United States).

Na^+^/K^+^-ATPase (NKA) activity is a sensitive indicator of ionoregulatory disruptions in fish gills following exposure to a variety of ENM formulations (Federici et al., 2007; Farmen et al., 2012; Schultz et al., 2012; Katuli et al., 2014; Bessemer et al., 2015; Black et al., 2017). NKA activity was measured in gill tissue from *F. diaphanus, A. agassizii*, and *P. axelrodi* by following the oxidation of NADH at 340 nm using a coupled colorimetric assay protocol (McCormick, 1993) and assuming an extinction coefficient of 6.22 mM^–1^ cm^–1^. Activities were normalized to total protein content using a DC protein assay (Bio-Rad Laboratories). Acetylcholinesterase (AchE) activity, which is a common target of xenobiotics and ENMs in fish (Katuli et al., 2014; Callaghan et al., 2016), was assayed in brain, gill, and heart tissue of *F. diaphanus* and in whole body homogenates of *A. agassizii* and *P. axelrodi* using the Ellman method (Ellman et al., 1961). Activity was calculated using an extinction coefficient of 13.6 mM^–1^ cm^–1^.

In all cases, absorbance was followed using either a Spectramax 190 or M2 microplate spectrophotometer (Molecular Devices, CA, United States).

### Data Analysis and Statistics

For whole animal studies, mass specific *ṀO_2_* was automatically calculated from decreases in dissolved O_2_ using the software associated with each respirometry system. In all species, *ṀO_2m__*in*_* was determined from a minimum of 3 representative *ṀO_2_* values taken from undisturbed fish in the final 2 h of each trial. In *F. diaphanus*, *ṀO_2m__*ax*_* represented the highest *ṀO_2_* value recorded following the exhaustive chase protocol and aerobic scope was calculated as the difference between *ṀO_2m__*in*_* and *ṀO_2m__*ax*_*. Variability in *ṀO_2_* recordings from undisturbed fish can be used as a proxy for spontaneous aerobic activity in fish (Campbell et al., 2019). Frequency distributions of *ṀO_2_* recordings from the final 5 h of each respirometry trial were generated using Prism 7 (GraphPad Software, Inc., CA, United States). Plots illustrate the number of *ṀO_2_* recordings from each species that fell within each 30 mg kg^–1^ h^–1^ bin. The frequency distributions thus provide a qualitative means to visualize variability in the aerobic activity of the animals over the selected recording period (Campbell et al., 2019). In all trials, *ṀO_2_* returned to baseline levels within the first 4 h after insertion into the respirometry system and fish were not disturbed during the period over which the frequency distribution analysis was examined.

*Oncorhynchus mykiss* hepatocyte *Ṁ*O_2_ data were analyzed using a two-way ANOVA followed by a Bonferroni *post hoc* test. Data from *F. diaphanus* studies were analyzed by one-way ANOVA to assess differences between control, BA, and nB_2_O_3_ treatment groups. When significance was detected, Tukey *post hoc* tests were used for multiple comparisons. Data from the two Amazonian species were analyzed with two-tailed Student’s *t*-tests. *P* < 0.05 was used to indicate statistical significance. Box and whisker plots display the range and median values of the data set while all other data is presented as the mean ± standard error of the mean (SEM).

## Results

### nB_2_O_3_ Characterization

**FIGURE 1:**
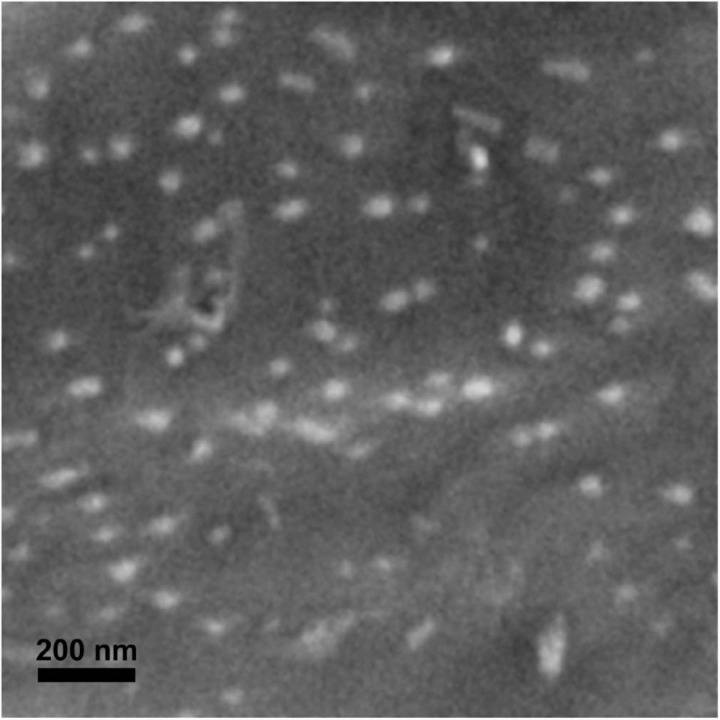
Scanning electron microscopy image of dry nB_2_O_3_.

The low atomic density of B_2_O_3_ prevented acquisition of high resolution images with the available instrument but images were sufficient for basic characterization purposes. Dry nB_2_O_3_ were spherical or oblong in shape with a mean primary particle diameter of 36.2 ± 1.4 nm (*n* = 20; Figure 1). Spherical particles ranged from 17 to 60 nm, while several higher aspect ratio particles were ∼125 × 50 nm. Dynamic light scattering illustrated hydrodynamic diameters of 189 ± 9 nm in *F. diaphanus* exposure water and 368 ± 43 nm in *P. axelrodi* and *A. agassizii* exposure water. Some degree of agglomeration thus occurred in all exposure scenarios and it was more evident in Amazonian water. This interpretation is supported by polydispersity indices of 0.230 for nB_2_O_3_ in *F. diaphanus* exposure water and 0.348 in Amazonian water (*P. axelrodi* and *A. agassizii* exposures). The DOC content of *F. diaphanus* exposure water determined here was 2.14 ± 0.55 and 0.8 mg L^–1^ in the Amazonian water (Sadauskas-Henrique et al., 2016). The higher DOC content of *F. diaphanus* exposure water may act to stabilize nB_2_O_3_ suspensions and limit agglomeration. ζ-potential was -17.2 ± 2.0 and -20.3 ± 1.2 mV for *F. diaphanus* exposure water and *P. axelrodi* and *A. agassizii* exposure water, respectively. The pH of the Amazonian water was 5.5 and nB_2_O_3_ had no effect on pH at concentrations up to 100 mg L^–1^. The pH of *F. diaphanus* exposure water was 7.5 and 100 mg L^–1^ nB_2_O_3_ increased it by 0.5 units.

### *Ṁ*O_2_ of Rainbow Trout Hepatocytes Exposed to BA and nB_2_O_3_

**FIGURE 2:**
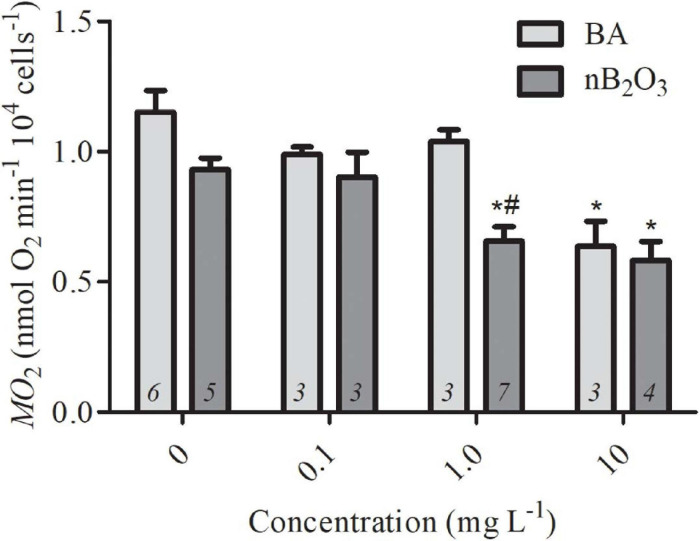
Rates of oxygen consumption (*Ṁ*O_2_) of *Oncorhychus mykiss* hepatocytes exposed to varying concentrations of BA and nB_2_O_3_. Data are presented as mean ± SEM and sample size is indicated within each bar. * Indicates significant difference from the corresponding control group and # indicates significant difference from BA-treated group of the same concentration.

The bioactivity of nB_2_O_3_ has not been well-characterized so a lowest observable effect concentration was first established in isolated *O. mykiss* hepatocytes and compared to BA to assess potential nano-specific effects (Figure 2). Both treatment (*p* = 0.0025) and concentration (*p* < 0.0001) significantly impacted hepatocyte *Ṁ*O_2_ and no interaction was noted (Figure 2). nB_2_O_3_ significantly reduced cellular *Ṁ*O_2_ at 1.0 mg L^–1^ relative to both the BA and control groups (*p* < 0.01). The *Ṁ*O_2_ of both treatment groups at 10 mg L^–1^ were lower than those of the corresponding control groups (both *p* < 0.01) but were not significantly different from one another. Based on these data, exposure concentrations of 1.0 and 10 mg L^–1^ were assigned for nB_2_O_3_ and BA, respectively, and used for subsequent whole animal studies on *F. diaphanus*.

### *Paracheirodon axelrodi* and *Apistogramma agassizii* nB_2_O_3_ 48 h LC_50_ Tests

During 48 h LC_50_ testing with nB_2_O_3_, two *P. axelrodi* died at the highest exposure concentration of 100 mg L^–1^ (data not shown). No other mortalities were noted in either species so an LC_50_ for nB_2_O_3_ could not be calculated. Sub-lethal effects were obvious at the highest doses; at 100 mg L^–1^ nB_2_O_3_, almost all fish (both species) lost equilibrium ∼30 min after the initial addition of nB_2_O_3_ and subsequently recovered several hours later. A mass loss of equilibrium was again observed after the 50% water change 24 h into the exposure and the animals again recovered several hours later. At the next highest dose of 10 mg L^–1^, fish of both species were clearly lethargic relative to untreated controls. As our focus was on potential sub-lethal effects of nB_2_O_3_, follow-up studies on *P. axelrodi* and *A. agassizii* employed an exposure dose of 10 mg L^–1^.

### Effects on Whole Animal and Tissue Energy Metabolism

**FIGURE 3:**
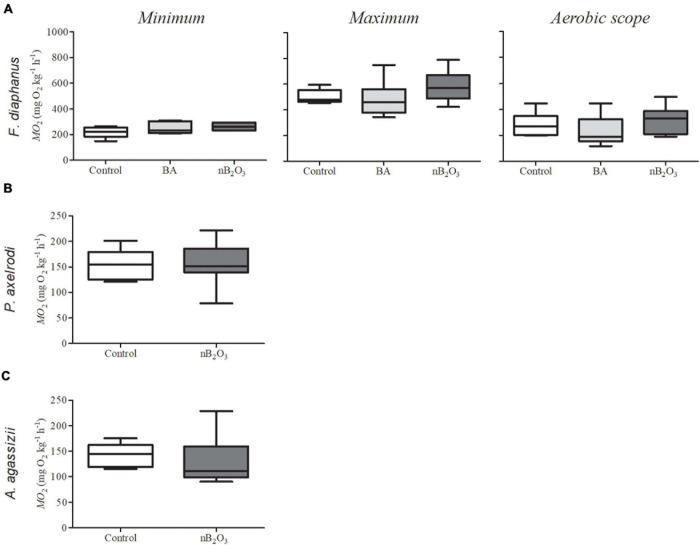
Rates of O_2_ consumption (*Ṁ*O_2_) in fish exposed for 48 h to boric acid (BA), nB_2_O_3_, or control conditions (*n* = 6–9). **(A)** Minimum and maximum *Ṁ*O_2_ and aerobic scope of *Fundulus diaphanus* exposed to 10 mg L^–1^ BA, 1.0 mg L^–1^ nB_2_O_3_, or control conditions (*n* = 6 for all conditions). **(B)** Minimum *ṀO_2_* in *Paracheirodon axelrodi* exposed to 10 mg L^–1^ nB_2_O_3_ or control conditions (*n* = 8 for both) and **(C)** *Apistogramma agassizii* exposed to 10 mg L^–1^ nB_2_O_3_ (*n* = 9) or control conditions (*n* = 7).

Loss of equilibrium and/or lethargy was not noted in *F. diaphanus* immediately following nB_2_O_3_ addition but, objectively, this species is less active than the Amazonian species and studies were carried out in opaque holding tanks which precluded accurate behavioral observations. In *F. diaphanus*, whole animal *Ṁ*O_2m__*in*_, *Ṁ*O_2m__*ax*_, and aerobic scope were not affected by a 48 h BA (10 mg L^–1^) or nB_2_O_3_ (1.0 mg L^–1^) exposure (Figure 3A). *Ṁ*O_2m__*in*_ was similarly unaffected in *P. axelrodi* and *A. agassizii* exposed to nB_2_O_3_ (10 mg L^–1^) for 48 h (Figures 3B,C, respectively).

**FIGURE 4:**
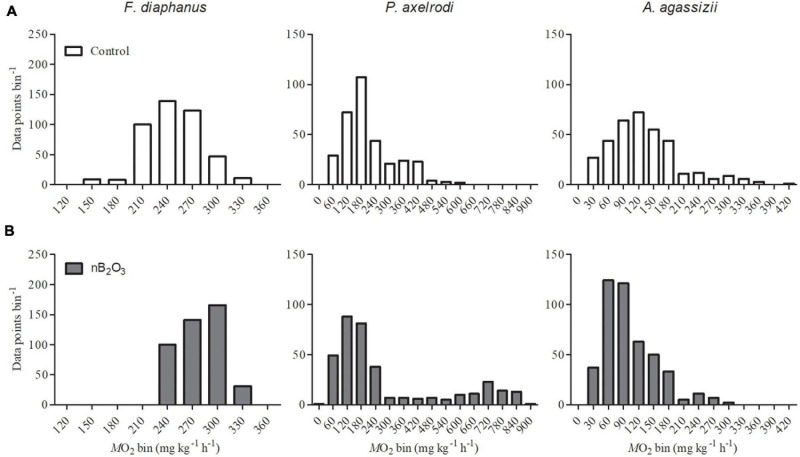
Frequency distributions of whole animal *ṀO_2_* recordings (*n* = 6–9, as specified in Figure 3) were used as a proxy for spontaneous aerobic activity in *Fundulus diaphanus, Paracheirodon axelrodi*, and *Apistogramma agassizii* exposed to control conditions **(A)** or to nB_2_O_3_ (**B**; 1.0 mg L^–1^ for *F. diaphanus* and 10 mg L^–1^ for *P. axelrodi* and *A. agassizii*). Data were collected from undisturbed fish over the final 5 h of each *ṀO_2_* recording.

Qualitatively, spontaneous aerobic activity in undisturbed fish appeared similar between control and nB_2_O_3_ treatment groups in all species, with the possible exception of *P. axelrodi* (Figure 4). *Ṁ*O_2_ was very consistent and stable in *F. diaphanus*, highly variable in *P. axelrodi*, and intermediate in *A. agassizii*. nB_2_O_3_ treated *P. axelrodi* exhibited periods of elevated aerobic activity not evident in control fish, reaching 5–6-fold above *Ṁ*O_2m__*in*_.

In *F. diaphanus*, there were no significant differences in liver glucose levels among treatment groups (Table 1). Both BA and nB_2_O_3_ treated *F. diaphanus* exhibited significant hepatic lactate accumulation relative to controls (*p* = 0.001) and nB_2_O_3_ treated fish accumulated more lactate than BA treated animals (Table 1). Whole body lactate levels were low and unaffected by nB_2_O_3_ treatment in the two Amazonian species.

**TABLE 1:** Effects of BA (10 mg L^–1^) and nB_2_O_3_ (1.0 mg L^–1^ for *Fundulus diaphanus*, 10.0 mg L^–1^ for *Paracheirodon axelrodi*, and *Apistogramma agassizii*) exposure on biomarkers of energy metabolism and oxidative stress.

**Biomarker**	**Species**	**Tissue**	**Treatment**		
			**Control**	**BA**	**nB_2_O_3_**
**Energy metabolism**					
Glucose (μmol g tissue^–1^)	*F. diaphanus*	Liver	24.56 ± 1.87 (6)	19.83 ± 4.11 (6)	21.55 ± 2.01 (6)
Lactate (μmol g tissue^–1^)	*F. diaphanus*	Liver	11.18 ± 1.22^*a*^ (9)	16.36 ± 0.80^*b*^ (9)	20.38 ± 2.17^*c*^ (9)
	*P. axelrodi*	W.B.	0.67 ± 0.09 (6)		0.57 ± 0.07 (6)
	*A. agassizii*	W.B.	0.96 ± 0.22 (6)		1.51 ± 0.22 (6)
**Oxidative stress**					
G6PDH (μmol min^–1^ mg protein^–1^)	*F. diaphanus*	Liver	0.19 ± 0.02 (3)	0.18 ± 0.03 (3)	0.14 ± 0.05 (3)
GR (nmol min^–1^ mg protein^–1^)		Liver	35.98 ± 7.90 (3)	38.28 ± 5.37 (3)	24.98 ± 7.51 (3)
MDA (μmol mg protein^–1^)		Liver	1.53 ± 0.42 (3)	1.59 ± 0.32 (3)	1.77 ± 0.38 (3)
LPO (μmol L^–1^ cumene H_2_O_2_ mg protein^–1^)	*P. axelrodi*	W.B.	46.87 ± 5.70 (6)		55.65 ± 8.84 (6)
	*A. agassizii*	W.B.	59.21 ± 7.61 (6)		57.36 ± 6.37 (6)
GST (μmol min^–1^ mg protein^–1^)	*P. axelrodi*	W.B.	1.46 ± 0.20 (6)		1.43 ± 0.21 (6)
	*A. agassizii*	W.B.	1.35 ± 0.68 (6)		0.78 ± 0.10 (6)

*WB, whole body. Sample size is indicated in parentheses and statistically significant differences between treatment groups are indicated by dissimilar letters.*

### Indictors of Oxidative Stress and Toxicity

A number of oxidative stress indicators were assessed in the 3 study species used for *in vivo* exposures (Table 1). Activities of anti-oxidant defense enzymes in *F. diaphanus* were unchanged following exposure to BA or nB_2_O_3_ and a similar lack of response was noted for *P. axelrodi*, and *A. agassizii*. Lipid peroxidation products, which are indicative of oxidative damage, did not vary significantly in any of the species examined.

**FIGURE 5:**
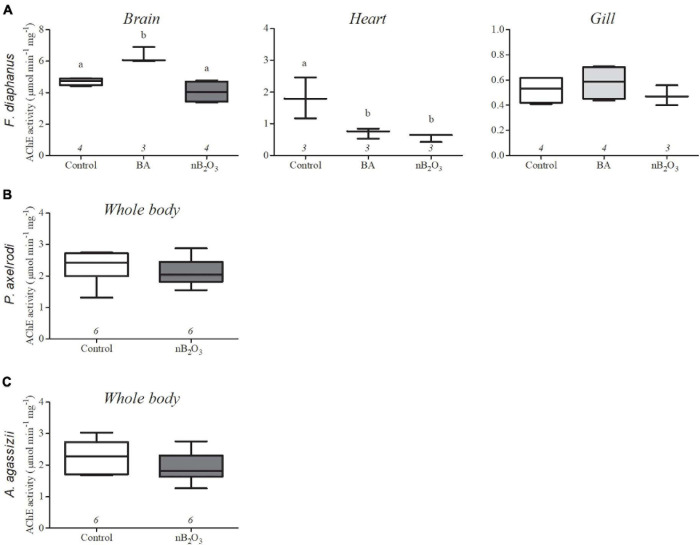
Effects of BA (10 mg L^−1^) and nB_2_O_3_ [1.0 mg L^−1^ for *Fundulus diaphanus* **(A)**, 10.0 mg L^−1^ for *Paracheirodon axelrodi* **(B)**, and *Apistogramma agassizii* **(C)**] exposure on tissue acetylcholinesterase (AChE) activity. Sample size is indicated beneath each plot and significant differences between treatment groups are denoted by dissimilar letters.

In *F. diaphanus*, brain AChE activity was significantly impacted by treatment (*p* = 0.0012) and increased in response to BA but not to nB_2_O_3_ (Figure 5). Heart AChE activity was also significantly affected by treatment (*p* = 0.0159), with inhibition by both BA and nB_2_O_3_, while activity was unaffected in gill. Whole body AChE activities were not altered by nB_2_O_3_ exposure in either *P. axelrodi* or *A. agassizii*.

**FIGURE 6:**
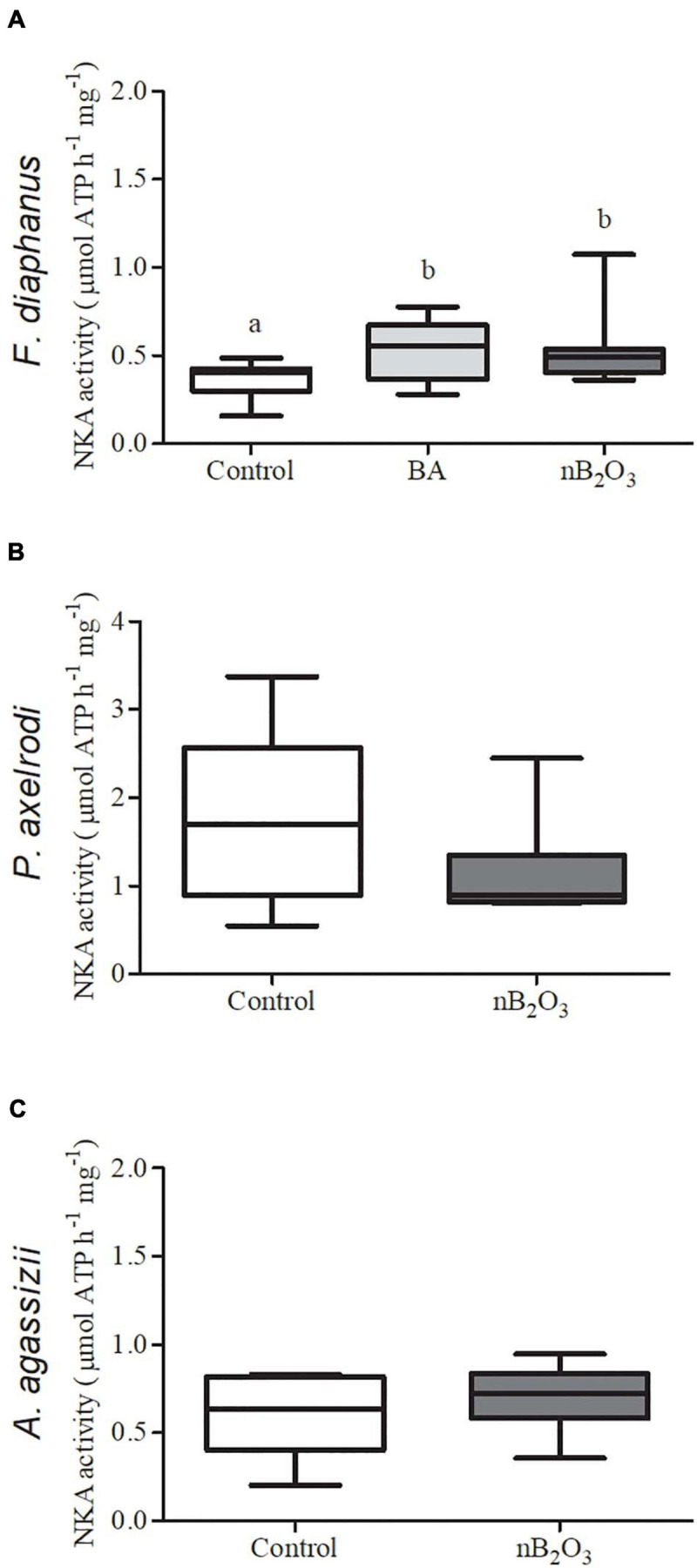
Effects of BA (10 mg L^−1^) and nB_2_O_3_ [1.0 mg L^−1^ for *Fundulus diaphanus* **(A)**, 10.0 mg L^−1^ for *Paracheirodon axelrodi* **(B)**, and *Apistogramma agassizii* **(C)**] exposure on gill Na^+^/K^+^-ATPase (NKA) activity (*n* = 12 in each condition for *F. diaphanus* and 6 in each condition for *P. axelrodi* and *A. agassizii*). Significant differences between treatment groups are denoted by dissimilar letters.

Gill NKA activity in *F. diaphanus* was significantly altered by treatment (*p* = 0.0234), with exposure to either BA or nB_2_O_3_ modestly increasing activity relative to controls (Figure 6). *Paracheirodon axelrodi* exhibited the highest gill NKA activity of the 3 species assessed but activity was not affected by nB_2_O_3_ exposure in either it or *A. agassizii*.

## Discussion

Increased industrial use of nB_2_O_3_ will inevitably lead to their release into the environment but their potential impacts on non-target organisms are almost entirely unknown. The natural form of boron (BA) is considered essentially non-toxic to vertebrates (Woods, 1994), but it is difficult to predict if bioactivity will also be low in nanoparticulate boron formulations. We addressed this gap in knowledge by examining cellular, whole animal, and biochemical indicators of nB_2_O_3_ toxicity in 3 fish species known to exhibit differing sensitivities to contaminants. Bioactivity was evident at all levels of biological organization examined and species-specific responses were observed, but only at relatively high exposure doses.

### nB_2_O_3_ Effects on Metabolism, Ionoregulation, and Neurotransmission Are Species-Specific

In rainbow trout hepatocytes, nB_2_O_3_ was bioactive at a lower concentration than BA, with *ṀO_2_* decreased at exposure doses of 1.0 vs. 10 mg L^–1^, respectively. The mechanism(s) underlying *ṀO_2_* inhibition were not assessed but boron can interact with a variety of enzymes and cofactors (Woods, 1994), which could subsequently reduce aerobic metabolic demand or trigger cell death. The high exposure doses required to elicit hepatocyte responses suggest that neither nB_2_O_3_ nor BA are particularly toxic, at least for this cell type.

Some ENM formulations specifically alter *ṀO_2m__*in*_* or *ṀO_2m__*ax*_* in fish (Black et al., 2017; Campbell et al., 2019), while in other species, subtle effects on both contribute to reductions in aerobic scope (Callaghan et al., 2016). The aerobic metabolic depression induced by nB_2_O_3_ in isolated hepatocytes was not replicated in whole animal exposures with any species tested. However, hepatocyte *ṀO_2_* was measured immediately following nB_2_O_3_ addition, while whole animal respirometry trials were only carried out following 48 h of nB_2_O_3_ exposure. The initial loss of equilibrium and lethargy we witnessed immediately after nB_2_O_3_ exposure in the 2 Amazonian species could be associated with a decrease in *ṀO_2_*, but additional studies are necessary to confirm this short-term effect of nB_2_O_3_ on aerobic metabolism of both species. In white sucker, *Catostomus commersonii*, zinc oxide ENM exposure (1.0 mg L^–1^) triggers a temporary decrease in heart rate (Bessemer et al., 2015) and reduces aerobic scope (Callaghan et al., 2016). We speculated that this related to a disruption of parasympathetic control of the cardiorespiratory system, either via ENM interactions with gill chemoreceptors or through direct inhibition of cardiac AChE activity, which led to a pseudo-hypoxic response. In white sucker, zinc oxide ENMs inhibited cardiac AChE activity and enhanced gill NKA activity (Bessemer et al., 2015; Callaghan et al., 2016), the latter likely resulting from an increase in gill blood flow. Inhibition of cardiac AChE and increased gill NKA were also observed here in *F. diaphanus* exposed to nB_2_O_3_. It is possible that acute nB_2_O_3_ exposure may trigger a similar, temporary pseudo-hypoxic response in some fish species, although more data is needed to support this supposition.

The *ṀO_2_* variability analysis indicated that after 48 h exposure, spontaneous activity might increase in nB_2_O_3_ treated *P. axelrodi*, but not in the other species tested. The highly variable *ṀO_2_* data from *P. axelrodi* may be related to its biology; *P. axelrodi* is an active species that is highly dependent on aerobic metabolism, and the large increases in *ṀO_2_* may be associated with a stress response. This species is likely to exploit aerobic activity in an attempt to escape the nB_2_O_3_ contaminated environment. In contrast, *A. agassizii* and *F. diaphanous* are less active species and may be more resilient to stressors. This same stress pattern was seen for *P. axelrodi* whereby *ṀO_2m__*in*_* increased almost threefold when fish were acutely exposed to 45 μg L^–1^ copper for 24–72 h, while no alterations were observed in *A. agassizii* (Braz-Mota et al., 2018). Such a response may indicate that *P. axelrodi* is more vulnerable to nB_2_O_3_ exposure under natural conditions, as such additional aerobic demands would leave less scope available for activities such as foraging, reproduction, or predator avoidance.

Gill Na^+^ uptake mechanisms differ between *A. agassizii* and *P. axelrodi* and may contribute to their distinct (putative) behavioral responses to nB_2_O_3_, although no differences in gill NKA activity were noted in either species in response to nB_2_O_3_ exposure. Although qualitative, these findings, along with the temporary loss of equilibrium of both Amazonian species at high exposure doses, warrant further investigation. Other ENM formulations significantly alter fish behavior with few other signs of overt toxicity (Botha et al., 2019) and these effects may translate to greater population-level impacts than changes in biochemical markers of toxicity alone (Scott and Sloman, 2004).

Exposure to BA or nB_2_O_3_ triggered significant liver lactate accumulation in *F. diaphanus*, while nB_2_O_3_ had no effect on whole body lactate in *P. axelrodi* or *A. agassizii*. If lactate accumulation was restricted to liver in *P. axelrodi* or *A. agassizii*, it may not have been detected in whole body homogenates. Glucose levels were not affected in any species under the conditions tested. Lactate usually accumulates in the absence of sufficient oxygen, but disruption of a metabolic pathway could also cause such a response. In the liver of white sucker, zinc oxide ENMs oxidatively inhibits aconitase (Dieni et al., 2014), the enzyme responsible for isomerizing citrate to isocitrate in the tricarboxylic acid (TCA) cycle. Decreased activity of this enzyme could theoretically inhibit flux through the TCA cycle and trigger the activation anaerobic metabolism even in the presence of abundant oxygen. This explanation is not well supported however, since we found no evidence of oxidative stress (see below). The observed increase in gill NKA activity in *F. diaphanus* hints at the possibility that BA or nB_2_O_3_ may have damaged the gill epithelium. If this occurred, impaired O_2_ uptake and hypoxemia may trigger hepatic lactate accumulation. This explanation is equally dubious though, as *ṀO_2m__*ax*_*, which is dependent upon a functional O_2_ transport cascade, was not affected by BA or nB_2_O_3_ in *F. diaphanus*.

Gill NKA activity in fish is impacted by exposure to a variety of ENM formulations (Federici et al., 2007; Farmen et al., 2012; Schultz et al., 2012; Katuli et al., 2014; Bessemer et al., 2015; Black et al., 2017). Both BA and nB_2_O_3_ exposure significantly stimulated gill NKA activity in *F. diaphanus*, but nB_2_O_3_ exposure had no effect on activity in either Amazonian species. ENM-induced gill membrane damage may also indirectly stimulate NKA activity (Bessemer et al., 2015), but the maintenance of *ṀO_2m__*in*_* and *ṀO_2m__*ax*_* suggests the gill is healthy, at least with respect to its respiratory functions. As mentioned above, it is possible that an increase in gill blood flow resulting from pseudo-hypoxic response could enhance diffusive Na^+^ losses to the environment, stimulating NKA activity as a compensatory response to maintain Na^+^ balance in this species. Boron also interacts with vertebrate membranes and alters their biophysical properties (Verstraeten et al., 2005), which may affect NKA function. The molecular activity of NKA increases as lateral membrane pressures increase (Wu et al., 2004), so it is possible that boron stimulated NKA activity by altering the structure of the gill basolateral membrane. Clearly, more work is necessary to confidently assign a mechanism to this observation and to evaluate the potential of BA and nB_2_O_3_ to disrupt Na^+^ homeostasis in *F. diaphanous*.

### nB_2_O_3_ Exposure Does Not Result in Oxidative Stress

Oxidative stress is a common mechanism of toxicity across a wide variety of ENM formulations and is frequently observed in ENM-exposed fish (Handy et al., 2011; Callaghan and MacCormack, 2017). The activities of G6PDH and GR were examined in liver tissue of *F. diaphanus* and GST activity was assessed in whole body homogenates of *P. axelrodi* or *A. agassizii*; these enzymes play important roles in the defense against oxidative stress (Tian et al., 1999; Hellou et al., 2012). G6PDH produces the NADPH required by GR to reduce oxidized glutathione, which is a key anti-oxidant molecule responsible for scavenging ROS, while GST conjugates reduced glutathione to xenobiotics to defend against oxidative stress. None of these enzymes were impacted by nB_2_O_3_ exposure, nor were LPO or MDA concentrations, which are biomarkers of oxidative damage. Under the conditions tested, nB_2_O_3_ exposure does not illicit a significant oxidative stress response in any of the species tested.

### Bioactivity Is Similar Between Boric Acid and nB_2_O_3_

In most instances, BA and nB_2_O_3_ exposure triggered similar biological responses, with a few exceptions. In isolated rainbow trout hepatocytes, nB_2_O_3_ impacted cellular *ṀO_2_* at a lower exposure concentration than BA but this effect did not translate to *in vivo ṀO_2_* responses in *F. diaphanus*, where neither compound had an effect. At a lower exposure concentration, nB_2_O_3_ also triggered a significantly greater accumulation of lactate in *F. diaphanus* but the general response to BA was still similar. Lastly, BA significantly increased brain AChE activity, while nB_2_O_3_ had no effect. These subtle differences in bioactivity could relate to the relative bioavailability of each compound at the target site of action. Cellular boron uptake occurs via specific transporters (Ocampo-Néstor et al., 2017) while ENMs are often internalized and processed via the lysosomal system (Wang et al., 2013). It is unclear how these mechanisms may impact the downstream bioavailability and bioactivity of BA and nB_2_O_3_. Overall, there are few clear nano-specific effects of nB_2_O_3_ but the bioactivity that is noted is realized at lower exposure doses than BA.

## Conclusion

nB_2_O_3_ is one of a number of boron ENM formulations with potential industrial, consumer, and clinical applications but little information is available on their potential ecotoxicity. Our results illustrate that nB_2_O_3_ exhibits species-specific bioactivity toward north-temperate and Amazonian freshwater fish. To our knowledge, environmental nB_2_O_3_ concentrations have not been studied, but based on modeling for other ENM formulations (Gottschalk et al., 2009; Keller and Lazareva, 2014), the exposure doses required to generate bioactivity (1–10 mg L^–1^) likely far exceeded environmental relevance. A lower nB_2_O_3_ exposure (0.1 mg L^–1^) dose had no effect on cellular *ṀO_2_* in hepatocytes but additional concentrations should be assessed in whole animal exposures to rule out the possibility of a hormetic response, which is observed with other ENM formulations (Iavicoli et al., 2014).

## Data Availability Statement

The raw data supporting the conclusions of this article will be made available by the authors, without undue reservation.

## Ethics Statement

The animal study was reviewed and approved by the Mount Allison University Animal Care Committe.

## Author Contributions

All authors listed have made a substantial, direct and intellectual contribution to the work, and approved it for publication

## Conflict of Interest

The authors declare that the research was conducted in the absence of any commercial or financial relationships that could be construed as a potential conflict of interest.
